# Effects of obesity with reduced 25(OH)D levels on bone health in elderly Chinese people: a nationwide cross-sectional study

**DOI:** 10.3389/fimmu.2023.1162175

**Published:** 2023-04-27

**Authors:** Chunchun Yuan, Jing Wang, Weiqiang Zhang, Honggang Yi, Bing Shu, Chenguang Li, Qianqian Liang, De Liang, Bolai Chen, Xingwen Xie, Xinchao Lin, Xu Wei, Hui Wang, Peizhan Chen, Chen Huang, Hao Xu, Yueli Sun, Yongjian Zhao, Qi Shi, Dezhi Tang, Yongjun Wang

**Affiliations:** ^1^ Longhua Hospital, Shanghai University of Traditional Chinese Medicine, Shanghai, China; ^2^ Spine Institute, Shanghai University of Traditional Chinese Medicine, Shanghai, China; ^3^ Key Laboratory of Theory and Therapy of Muscles and Bones, Ministry of Education (Shanghai University of Traditional Chinese Medicine), Shanghai, China; ^4^ Shanghai Geriatric Institute of Chinese Medicine, Shanghai, China; ^5^ Academic Research Center of Shixiaoshan’ Traumatology, Shanghai, China; ^6^ State Key Laboratory of Genetic Engineering, Collaborative Innovation Center for Genetics and Development, School of Life Sciences and Human Phenome Institute, Fudan University, Shanghai, China; ^7^ Department of Biostatistics, School of Public Health, Nanjing Medical University, Nanjing, China; ^8^ The First Affiliated Hospital of Guangzhou University of Chinese Medicine, Guangzhou, China; ^9^ Guangdong Provincial Hospital of Chinese Medicine, Guangzhou, China; ^10^ The Second Affiliated Hospital of Guangzhou University of Chinese Medicine, Guangzhou, China; ^11^ The Second People’s Hospital of Gansu Province, Gansu, Lanzhou, China; ^12^ Affiliated Hospital of Northwest University for Nationalities, Gansu, Lanzhou, China; ^13^ Dongzhimen Hospital, Beijing University of Chinese Medicine, Beijing, China; ^14^ Wangjing Hospital, China Academy of Chinese Medical Sciences, Beijing, China; ^15^ State Key Laboratory of Oncogenes and Related Genes, Center for Single-Cell Omics, School of Public Health, Shanghai Jiao Tong University School of Medicine, Shanghai, China; ^16^ Shanghai Institute of Digestive Surgery, Ruijin Hospital, Shanghai Jiao Tong University School of Medicine, Shanghai, China

**Keywords:** osteoporosis, older people, obesity, reduced 25(OH)D, cohort study

## Abstract

**Background:**

Obesity is often accompanied by lower 25(OH)D levels, whereas these two parameters exhibit opposite effects on bone health. It is uncertain what are the effects of lower 25(OH)D levels in obesity on bone health in elderly Chinese people.

**Methods:**

A nationally representative cross-sectional analysis of China Community-based Cohort of Osteoporosis (CCCO) was performed from 2016 to 2021, which consisted of 22,081 participants. Demographic data, disease history, Body mass index (BMI), bone mineral density (BMD), the levels of the biomarkers of vitamin D status and those of bone metabolism markers were measured for all participants (N = 22,081). The genes (rs12785878, rs10741657, rs4588, rs7041, rs2282679 and rs6013897) related to 25(OH)D transportation and metabolism were performed in a selected subgroup (N = 6008).

**Results:**

Obese subjects exhibited lower 25(OH)D levels (p < 0.05) and higher BMD (p < 0.001) compared with those of normal subjects following adjustment. The genotypes and allele frequency of rs12785878, rs10741657, rs6013897, rs2282679, rs4588 and rs7041 indicated no significant differences among three BMI groups following correction by the Bonferroni’s method (p > 0.05). The levels of total 25(OH)D (ToVD) were significantly different among the GC1F, GC1S and GC2 haplotype groups (p < 0.05). Correlation analysis indicated that ToVD levels were significantly correlated with parathyroid hormone levels, BMD, risk of osteoporosis (OP) and the concentration levels of other bone metabolism markers (p < 0.05). Generalized varying coefficient models demonstrated that the increasing BMI, ToVD levels and their interactions were positively associated with BMD outcomes (p < 0.001), whereas the reduced levels of ToVD and BMI increased the risk of OP, which was noted notably for the subjects with reduced ToVD levels (less than 20.69 ng/ml) combined with decreased BMI (less than 24.05 kg/m^2^).

**Conclusion:**

There was a non-linear interaction of BMI and 25(OH)D. And higher BMI accompanied by decreased 25(OH)D levels is associated with increased BMD and decreased incidence of OP, optimal ranges exist for BMI and 25(OH)D levels. The cutoff value of BMI at approximately 24.05 kg/m^2^ combined with an approximate value of 25(OH)D at 20.69 ng/ml are beneficial for Chinese elderly subjects.

## Introduction

Osteoporosis occurs mainly in the process of aging in populations over 50 years old and is referred as the decrease of bone mineral density (BMD) or imbalance between bone resorption and formation (1). Worldwide, approximately 200 million subjects have osteoporosis (2), which causes significant financial burden on the corresponding countries and higher health costs for families (3). BMD is detected using dual-energy X-ray absorptiometry (DXA) and is considered an effective index for evaluating bone health (4).

Body mass index (BMI) is usually used to assess bone health, since it can be determined conveniently and is not influenced by affections of other physical or pathological factors (5). Higher BMI was always regarded as a protective factor for increasing BMD (6), due to higher weight which could promote the increase in BMD (7, 8) and maintain myokine levels. The latter positively regulate bone formation and reabsorption (9, 10). However, certain studies have shown that populations with higher BMI suffer more easily from fractures (11). The optimal ranges of BMI may play a role in balancing the relationship between bone protection and bone fracture.

25(OH)D, including 25(OH)D_2_ and 25(OH)D_3_, is considered the marker of vitamin D (VD) status (12) and it has been shown that the lower a person’s VD level, the greater the likelihood of poor health, disease occurrence and mortality (13). Concomitantly, several studies have shown statistical correlations between lower 25(OH)D levels and decreasing BMD (14) as well as skeletal disorders including osteopenia, osteoporosis and the risk of fractures (15, 16).

BMI is inversely associated with 25(OH)D levels (17, 18), but it is still uncertain whether reduced 25(OH)D levels in subjects with higher BMI will attenuate bone health. In the present study, the interactive effects of 25(OH)D levels and BMI were evaluated on BMD and osteoporosis. The study aimed at providing the quantitative results in Chinese elderly subjects in order to guide the clinical diagnosis and treatment of bone-related diseases.

## Methods and analysis

### Study population

The Chinese community-based cohort of osteoporosis (CCCO) is a population-based nationwide, prospective cohort study, which has been ongoing since 2016 and it currently includes 22,081 participants (19). A cross-sectional study was performed in middle-aged and older participants who were living in urban areas from CCCO that were distributed in Shanghai (East China), Beijing (North China), Guangzhou (South China) and Lanzhou (West China). Only participants who had completed genotyping, and those with BMI > 18.5 kg/m^2^, and had no a history of endocrine and metabolic diseases, and were not taken VD, calcium, estrogen, diphosphonate and glucocorticoid analogues were included in the present study (4,124 participants) (**Figure S1** in the **Supplementary Materials**).

The present study, registered protocol at Clinical trials. gov (NCT02958020), was approved by the institutional review board at Longhua Hospital affiliated to the Shanghai University of Traditional Chinese Medicine (No. 2016LCSY065) and was implemented in accordance with the ethical standards laid down in the 1964 Declaration of Helsinki and its later amendments or comparable ethical standards. All subjects provided written informed consent.

### Data collection and measurements

Basic characteristics, disease history, physical measurements and other common information of the participants were collected in the community field work. An interviewer-administrator paper questionnaire was implemented by experienced physicians to facilitate a higher response rate. The International Physical Activity Questionnaire (IPAQ) was used to access the physical activity conditions. The data of the mean annual hours corresponding to sunshine were derived from the Chinese Meteorological Administration (http://cdc.cma.gov.cn/).

Weight and height were measured with the participants wearing no shoes and were used to define BMI. The World Health Organization criteria were used in the present study for the Chinese population and 3 BMI (kg/m^2^) categories were provided as follows: normal weight (18.5-22.9 kg/m^2^), overweight (22.9-24.9 kg/m^2^), and obese (>25.0 kg/m^2^) (20). The grip strength of both hands was determined with a hand dynamometer and five-times-sit-to-stand test (FTSST) was used to evaluate the physical function. Lumbar and hip BMD were measured with the DXA (Hologic Discovery CI, Bedford, Massachusetts, USA) equipped in a mobile research vehicle.

### Laboratory analyses

25(OH)D was measured with a Shimadzu series HPLC (Shimadzu Corporation, Japan) instrument connected to an API 5500 LC-MS/MS system (Applied Biosystems Inc., USA). Bioavailable 25(OH)D (BioVD) levels were calculated following the methods of Powe et al. (2013) (21). The plasma vitamin D binding protein (DBP) concentrations were measured with a highly sensitive two-site enzyme linked immunoassay (ELISA) test kit (GENWAY, BIOTECH INC, USA).

Parathyroid hormone (PTH), albumin (Alb), creatinine (CRE), calcium (Ca), phosphorous (P), alkaline phosphatase (ALP), N-terminal propeptide of type I procollagen (PINP), osteocalcin (OST) and β-Cross Laps of type I collagen levels containing cross linked C-telopeptide (CTX) were detected with a Roche automatic analyzer method (see the detailed methods of **Table S1** in the **Supplementary Materials**). Genotyping methods can be found in the **Supplementary Methods** and gene locus single nucleotide polymorphisms (SNPs) information is described in **Table S2** in the **Supplementary Materials**.

### Statistical analysis

We used a cross-sectional study design to explore the population-level association between the explanatory covariates and BMD outcomes. The continuous variables were summarized as mean (standard deviation) or median (inter-quartile range) depending on the normativity of the distribution. The categorical variables were listed as numbers and percentages. The box-cox transformation was used to reduce the skewness of the outcomes. The univariate analysis was performed by examining the differences of the BMI or genetic groups using the *χ* ^2^ test for categorical variables and the one-way ANOVA or Kruskal-Wallis test for continuous variables. Spearman’s rank correlation was used to assess the correlation among serum and BMD measures.

Generalized varying coefficient models (GVCM) with penalized cubic regression splines were employed to characterize the nonlinear relationships of outcomes and explanatory covariates, as well as the interactions of explanatory covariates to account for the complexity. The relationship between each covariate and the outcome is allowed to vary as a potentially nonlinear, smooth function of an effect modifying covariate estimated from the data in the GVCM. A stepwise forward method was used to select the variables in the final model for multivariate analysis. Bayesian 95% confidence intervals (CI) were estimated to present the association of each covariate with the outcome.

All results were calculated and adjusted for the identified potential confounders as fixed effects in the GVCMs, including age (in years, continuous), sex (men or women), educational status, smoking status, drinking status, dietary types, family income levels, seasons, regions, and mean annual hours of sunshine (in hours, continuous). Missing data patterns were visualized and imputed using the R multivariate imputation by the chained equation (MICE) package in R (the number of imputations is set to 5). The GVCM was fitted to each imputed dataset and the results were combined across the 5 datasets using the Rubin’s method, which computes imputation-adjusted variances and effects estimations.

All p values presented were two-sided, with statistical significance determined by a family-wise type I error less than 0.05. The Bonferroni’s method was used for multiple comparisons following multiple imputations. All the analyses were performed with the use of R software (R Foundation for Statistical Computing, version 4.0.5) and Stata 15.0 software (STATA Corp, College Station, TX). Additional details regarding the statistical analysis are provided in the Methods section of the **Supplementary Materials**.

## Results

### Characteristics of the participants

Obese (1,653 participants), overweight (1,039 participants) and normal (1,432 participants) were similar in terms of sampling seasons, dietary types and drinking habits (**Table 1**). However, three groups were different with regard to gender, age, BMI, mean annual hours of sunshine, family income, educational degree and smoking (**Table 1**). The genotyping results indicated that none of the six variants was departed from the Hardy-Weinberg equilibrium (HWE) test.

**Table 1:** Characteristics of included subjects in normal, overweight and obese groups (N = 4,124).

Characteristics	Normal (n = 1,432)^#^	Overweight (n = 1,039)^#^	Obese (n = 1,653)^#^	p^*^
**Gender (man/women)**	363/1069	341/698	519/1134	**< 0.001**
**Age, median (P25-P75, IQR, years)**	63 (58-68, 10)	63 (58-68, 10)	64 (59-69, 10)	**0.047**
**BMI, median (P25-P75, IQR, kg/m^2^)**	21.41 (20.36-22.27, 1.91)	24.00 (23.45-24.44, 0.99)	26.73 (25.27-28.57, 3.3)	**< 0.001**
MAHS (cities)				
1548.5 hours (Guangzhou)	699 (48.8%)	429 (41.3%)	582 (35.2%)	**< 0.001**
1764.4 hours (Shanghai)	523 (36.5%)	401 (38.6%)	635 (38.4%)	
2510 hours (Beijing)	94 (6.6%)	80 (7.7%)	182 (11.0%)	
2526.2 hours (Lanzhou)	116 (8.1%)	129 (12.4%)	254 (15.4%)	
Sampling seasons				
March-May	181(12.6%)	123(11.8%)	187(11.3%)	0.116
June-August	951(66.4%)	707(68.0%)	1169(70.7%)	
September-November	217(15.2%)	136(13.1%)	207(12.5%)	
December-February	83(5.8%)	73(7.0%)	90(5.4%)	
Family income (RMB/month)				
≤2999	302 (21.1%)	199 (19.2%)	418 (25.3%)	**0.004**
3000-4999	408 (28.5%)	297 (28.6%)	450 (27.2%)	
5000-7999	440 (30.7%)	343 (33.0%)	531 (32.1%)	
8000-11999	192 (13.4%)	136 (13.1%)	169 (10.2%)	
≥12000	52 (3.6%)	42 (4.1%)	53 (3.2%)	
Education degree				
Primary school or illiteracy	254 (17.8%)	213 (20.5%)	386 (23.4%)	**< 0.001**
Junior high school	377 (26.3%)	273 (26.3%)	508 (30.7%)	
Senior high school	601 (42.0%)	396 (38.1%)	572 (34.6%)	
College education	134 (9.4%)	105 (10.1%)	110 (6.7%)	
Bachelor or above	49 (3.4)	36 (3.5%)	51 (3.1%)	
Dietary type				
Meat priority	62 (4.3%)	58 (5.6%)	100 (6.0%)	0.138
Vegetable priority	371 (25.9)	250 (24.1%)	382 (23.1%)	
Mixed	963 (67.2%)	703 (67.7%)	1123 (67.9%)	
Smoking				
Never	1088 (76.0%)	748 (72.0%)	1190 (72.0%)	**0.048**
Active	160 (11.2%)	137 (13.2%)	216 (13.1%)	
Passive (close encounter)	124 (8.7%)	86 (8.3%)	140 (8.5%)	
Before	54 (3.8%)	63 (6.1%)	93 (5.6%)	
Alcohol				
Never	1273 (88.9%)	868 (83.5%)	1355 (82.0%)	0.083
Current	127 (8.9%)	133 (12.8%)	226 (13.7%)	
Before	24 (1.7%)	28 (2.8%)	58 (3.5%)	

BMI, body mass index; IQR, interquartile range; MAHS, mean annual hours sunshine. Data were expressed as median with IQR for continuous variables or *n* (%) for categorical variables. Totals do not add up to 100% because of rounding or missing data.

*p values were calculated by Chi-square test, Fisher’s exact method, or Kruskal-Wallis test. Bold indicates statistically significant < 0.05.

#Participants were divided into three groups based off BMI including the normal group (18.5-22.9 kg/m^2^), overweight group (22.9-24.9 kg/m^2^), and obese group (> 25.0 kg/m^2^).

### Obese subjects exhibit lower 25(OH)D and higher BMD

**Figure 1:**
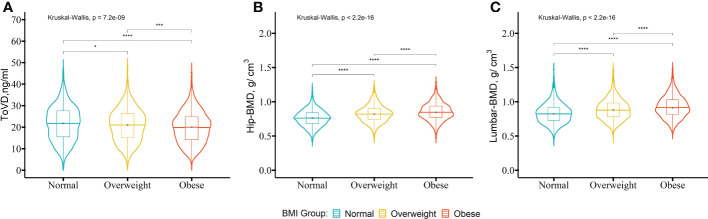
The levels of ToVD, Hip-BMD and Lumbar-BMD of the participants with different BMI groups. A comparison of the distributions of the biochemical parameters between the BMI groups was performed using the Kruskal-Wallis test. The *post-hoc* test was used in order to examine the differences between the pairs of the BMI groups with the Bonferroni’s method. The participants were divided into three groups based on BMI including the normal group (18.5-22.9 kg/m^2^), overweight group (22.9-24.9 kg/m^2^) and obese group (>25.0 kg/m^2^).The box represents the median and interquartile ranges. BMI, body mass index; ToVD, total 25(OH)D; Hip-BMD, hip bone mineral density; Lumbar-BMD, lumbar bone mineral density. ****p < 0.0001; ***p < 0.001; **p < 0.01; *p < 0.05.

The comparison among the normal, overweight and obese groups indicated that unadjusted levels of ToVD were gradually decreased statistically (normal, 21.80 ng/ml (IQ:15.60, 27.60 ng/ml); overweight 21.15 ng/ml (IQ:15.10, 26.45 ng/ml); obese, 20.05 ng/ml (IQ:14.40, 24.90 ng/ml), p < 0.001) (**Figure 1**). The differences between normal and obese groups persisted following multivariable adjustment (p < 0.05) (**Table S3** in the **Supplementary Materials**). The unadjusted median BMD of the hip was statistical greater in the higher BMI groups than that noted in the normal group (obese, 0.85 g/cm^3^ (IQ:0.77, 0.94 g/cm^3^) and overweight, 0.82 g/cm^3^ (IQ:0.74, 0.90 g/cm^3^) vs. normal, 0.76g/cm^3^ (IQ:0.68, 0.84 g/cm^3^), p < 0.001, respectively) (**Figure 1**). The significant differences of the three groups were applied to the median BMD of lumbar (obese, 0.91 g/cm^3^ (IQ: 0.81, 1.03 g/cm^3^) and overweight, 0.88 g/cm^3^ (IQ: 0.78, 0.98 g/cm^3^) vs. normal groups, 0.83g/cm^3^ (IQ: 0.73, 0.92 g/cm^3^), p < 0.001, respectively) (**Figure 1**). The same statistical differences in the hip and lumbar BMD persisted following multivariable adjustment (**Table S3** in the **Supplementary Materials**). The results without imputation were also consistent with the fore mentioned data (**Table S4** in the **Supplementary Materials**).

### Serum biomarkers and physical measurements of participants with different BMI

In the unadjusted and adjusted models, PTH was higher statistically in the obese group than that of the normal group (p < 0.001, p < 0.05, respectively) (**Table 2**). The expression levels of BioVD indicated statistical significance (p = 0.05), whereas no difference was noted between the groups following adjustment. Additional differences were not found following estimation of the proportion of BioVD. Albumin and DBP could associate with the concentration levels of BioVD. The levels of these two parameters did not differ according to the BMI value of each group. The p value was lower statistically in the overweight and obese groups than that noted in the normal group (p < 0.05, p < 0.001, respectively). There was no significant difference when specific parameters including season, region, sex, age, education, salary, smoking, drinking, diet and total annual sunshine hours were added to the model. Ca and CRE did not exhibit significant differences among the three groups investigated (**Table 2**).

**Table 2:** Comparision of serum biomarkers and physical measurements among different BMI participants.

	Normal^a^	Overweight^a^	Obese^a^	Unadjusted				Adjusted^c^			
				p value^b^	1 vs 2	1 vs 3	2 vs 3	p value^b^	1 vs 2	1 vs 3	2 vs 3
**PTH (pmol/L)**	3.00 (2.27, 3.97)	3.06 (2.34, 4.09)	3.30 (2.40, 4.35)	***<0.001***	0.254	***<0.001***	***0.010***	***0.015***	>0.99	***0.015***	0.199
**Alb (g/L)**	40.50 (32.90, 45.75)	41.30 (34.80, 45.70)	41.30 (35.00, 46.10)	0.086	0.644	0.083	>0.99	0.140	0.641	0.153	>0.99
**BioVD (ng/ml)**	1.24 (0.80, 1.97)	1.26 (0.76, 1.89)	1.18 (0.75, 1.85)	***0.050***	0.311	0.051	>0.99	0.697	>0.99	>0.99	>0.99
Percentage (%)^d^	6.11 (4.23, 8.91)	6.14 (4.33, 9.01)	6.27 (4.45, 8.89)	0.543	>0.99	>0.99	0.828	0.632	>0.99	>0.99	>0.99
**CRE (μmol/L)**	65.70 (58.80, 74.60)	66.90 (58.30, 77.10)	67.40 (58.70, 77.40)	0.492	>0.99	0.699	>0.99	0.537	0.810	>0.99	>0.99
**Ca (mmol/L)**	2.32 (2.27, 2.38)	2.32 (2.26, 2.38)	2.32 (2.26, 2.38)	0.223	0.969	0.256	>0.99	0.791	>0.99	>0.99	>0.99
**P (mmol/L)**	1.25 (1.09, 1.42)	1.21 (1.06, 1.40)	1.20 (1.03, 1.37)	***<0.001***	***0.021***	***<0.001***	0.067	0.297	>0.99	0.647	0.493
**DBP (μg/mL)**	424.08 (283.60, 645.96)	434.16 (299.00, 627.12)	445.98 (313.48, 639.86)	0.195	0.300	0.452	>0.99	0.182	0.269	0.444	>0.99
**ALP (U/L)**	72.00 (61.00, 85.00)	73.00 (63.00, 86.00)	75.00 (64.00, 89.00)	***0.007***	0.226	***0.005***	0.977	0.379	0.605	0.836	0.836
**PINP (ng/ml)**	51.53 (40.11, 64.95)	48.65 (38.49, 61.84)	48.21 (37.66, 61.64)	***<0.001***	***0.010***	***0.000***	>0.99	***0.007***	0.153	***0.005***	>0.99
**OST (ng/ml)**	16.56 (13.38, 20.55)	14.92 (11.97, 18.66)	13.91 (11.13, 17.34)	***<0.001***	***<0.001***	***<0.001***	***<0.001***	***<0.001***	***<0.001***	***<0.001***	***<0.001***
**CTX (ng/ml)**	0.31 (0.23, 0.41)	0.28 (0.22, 0.38)	0.28 (0.21, 0.38)	***<0.001***	***<0.001***	***<0.001***	0.248	***<0.001***	***0.003***	***<0.001***	***0.253***
**LGS (kg)**	22.70 (19.10, 27.20)	24.10 (20.00, 29.95)	23.70 (19.50, 30.30)	***<0.001***	***<0.001***	***0.001***	0.687	***0.019***	***0.044***	***0.050***	>0.99
**RGS (kg)**	24.20 (20.20, 28.70)	25.30 (20.80, 31.20)	25.00 (20.60, 31.50)	***<0.001***	***<0.001***	***<0.001***	>0.99	***0.022***	0.233	***0.019***	>0.99
**FTSST (s)**	7.50 (6.14, 9.07)	7.89 (6.33, 9.70)	8.10 (6.70, 10.00)	***<0.001***	***0.007***	***<0.001***	***0.002***	***0.002***	0.170	***0.001***	0.543
**PA (MET-min/w)**	2079.00 (1386.00, 3066.00)	1680.00 (1386.00, 3066.00)	1866.00 (1386.00, 3159.00)	0.142	0.184	>0.99	0.427	0.121	0.158	>0.99	0.313

1, Normal group; 2, Overweight group; 3, Obese group. PTH, parathyroid hormone; Alb, Albumin; BioVD, bioavailable 25(OH)D; CRE, Creatinine; Ca, Calcium; P, Phosphorous; DBP, vitamin D binding protein; ALP, alkaline phosphatase; PINP, N-terminal propeptide of type I procollagen; OST, osteocalcin; CTX, β-Cross Laps of type I collagen containing crosslinked C-telopeptide; LGS, left grip strength; RGS, right grip strength; FTSST, five-times-sit-to-stand test; PA, physical activity.

a Descriptive results were shown as the medians (interquartile range) of the original data.

b p values were estimated by multiple linear regression models using multiple imputations (the number of imputations was 5), and adjusted coefficients and standard errors for the variability between imputations according to the combination rules by Rubin. The significant p values were highlighted by bold, italics font. The skewed dependents variables were transformed using the Box-Cox transformation to approach normality for the linear regression model. Bonferroni’s adjustment for multiple comparisons were used for pairwise comparisons among groups after the multiple imputation estimations. The p values for pairwise comparisons were significant at the familywise error rate of 0.05 by Bonferroni’s adjustment.

c Adjusted by season, region, sex, age, education, salary, smoking, drinking, diet and mean annual hours sunshine.

d Percentage = BioVD/Total 25(OH)D×100%.

The comparison of bone metabolism markers among different groups demonstrated that PINP, CTX and OST indicated reduced levels in obese than those noted in normal subjects (p < 0.05, p < 0.001, p < 0.001, respectively, adjusted model). Although ALP levels were higher in obese subjects compared with those noted in normal subjects (p < 0.05), the results were altered due to adjustment. The grip strength of both hands was different between normal and obese groups following adjustment. FTSST was longer statistically in obese than in normal subjects (p < 0.001, p < 0.001, unadjusted and adjusted model). There is no significant difference among the three BMI groups on the level of PA (**Table 2**).

### Genetic polymorphisms, 25(OH)D levels and BMD

The classification of the genes according to BMI and ToVD can be found in **Figure S2** (**Supplementary Materials**), which indicates that haplotypes of the other types were not the same cluster as the Gc2, Gc1F and Gc1S. The genotype frequency and the allele frequency of the rs12785878, rs10741657, rs6013897, rs2282679, rs4588 and rs7041 SNPs showed no significant difference among the three BMI groups (**Table S5** in the **Supplementary Materials**). The frequency of the haplotype types was different significantly among groups (p < 0.05) (**Table S5** in the **Supplementary Materials**) and was sorted by the frequency of Gc2 from the highest to the lowest as follows: Obese, overweight and normal (**Figure S3** in the **Supplementary Materials**). ToVD levels were the lowest in Gc2 homozygous participants, intermediate in Gc1F homozygous participants and highest in Gc1S homozygous participants according to the unadjusted and adjusted models (**Table S6** in the **Supplementary Materials**). BMD levels did not differ significantly by the haplotype group (**Table S6** in the **Supplementary Materials**) and further analysis of osteoporosis indicated failure to support the correlation between gene and disease in the biological model of codominant, dominant and recessive inheritance after Bonferroni’s adjustment (**Table 3**).

**Table 3:** Association between single nucleotide polymorphisms and OP after multiple imputation.

Genotype		OP		Unadjusted^a^						Adjusted^a^					
				Codominant		Dominant		Recessive		Codominant		Dominant		Recessive	
		NO (n=2755)	Yes (n=1196)	OR	p	OR	p	OR	p	OR	p	OR	p	OR	p
**rs10741657**	**AA**	324	143	1.04(0.83-1.30)	0.7350	1.05(0.91-1.20)	0.5088	1.01(0.82-1.25)	0.8929	0.98(0.78-1.24)	0.1435	1.01(0.88-1.17)	0.8735	0.98(0.78-1.22)	0.8735
	**AG**	1186	528	1.05(0.91-1.21)	0.5138					1.02(0.88-1.18)	0.8735				
	**GG**	1233	524	ref = 1						ref = 1					
**rs12785878**	**GG**	937	421	1.18(0.96-1.44)	0.1084	1.17(0.98-1.41)	0.0813	1.05(0.91-1.21)	0.5006	1.17(0.95-1.45)	**0.0327**	1.15(0.95-1.39)	0.1435	1.07(0.92-1.24)	0.1435
	**GT**	1294	577	1.17(0.97-1.42)	0.1061					1.14(0.93-1.39)	**0.0208**				
	**TT**	517	197	ref = 1						ref = 1					
**rs2282679**	**GG**	254	108	0.91(0.71-1.16)	0.4541	0.86(0.75-0.98)	**0.0268**	0.98(0.78-1.24)	0.8734	0.89(0.69-1.15)	0.7314	0.85(0.73-0.97)	**0.0208**	0.97(0.76-1.24)	**0.0208**
	**GT**	1153	457	0.85(0.73-0.98)	**0.0225**					0.84(0.72-0.97)	0.3820				
	**TT**	1341	631	ref = 1						ref = 1					
**rs4588**	**TT**	277	121	0.96(0.76-1.21)	0.7271	0.89(0.77-1.02)	0.0838	1.02(0.82-1.28)	0.8511	0.95(0.74-1.21)	0.1435	0.86(0.74-0.99)	**0.0327**	1.03(0.81-1.3)	**0.0327**
	**TG**	1162	469	0.87(0.75-1.00)	0.0580					0.84(0.72-0.97)	0.8735				
	**GG**	1307	606	ref = 1						ref = 1					
**rs6013897**	**AA**	80	45	1.31(0.90-1.91)	0.1551	1.06(0.92-1.22)	0.4383	1.30(0.90-1.88)	0.1672	1.38(0.93-2.05)	**0.0327**	1.07(0.92-1.24)	0.3820	1.37(0.93-2.02)	0.3820
	**AT**	783	344	1.03(0.89-1.20)	0.6712					1.04(0.89-1.22)	**0.0208**				
	**TT**	1885	807	ref = 1						ref = 1					
**rs7041**	**GG**	246	120	1.12(0.88-1.42)	0.3557	1.02(0.89-1.17)	0.8077	1.12(0.89-1.41)	0.3263	1.13(0.88-1.45)	0.7314	1.03(0.89-1.18)	0.7314	1.13(0.89-1.43)	0.7314
	**TG**	1124	484	0.99(0.86-1.15)	0.9398					1.00(0.86-1.17)	0.3820				
	**TT**	1377	592	ref = 1						ref = 1					

OP, osteoporosis. *n* represents number of subjects. OR, odds ratio. 95%CI: 95% confidence interval. ref, reference level.

Totals do not add up to 100% because of rounding or missing.

a OR with 95%CI and p values were calculated by univariate and multivariate logistic regression after multiple imputations (the number of imputation is 5). The adjusted covariates, including season, sex, age, education, salary, smoking, drinking, diet and mean annual hours sunshine were adjusted in multivariate analysis. Bold means statistically significant < 0.05.

### Correlations between markers of vitamin D status and bone metabolism markers

**Figure 2:**
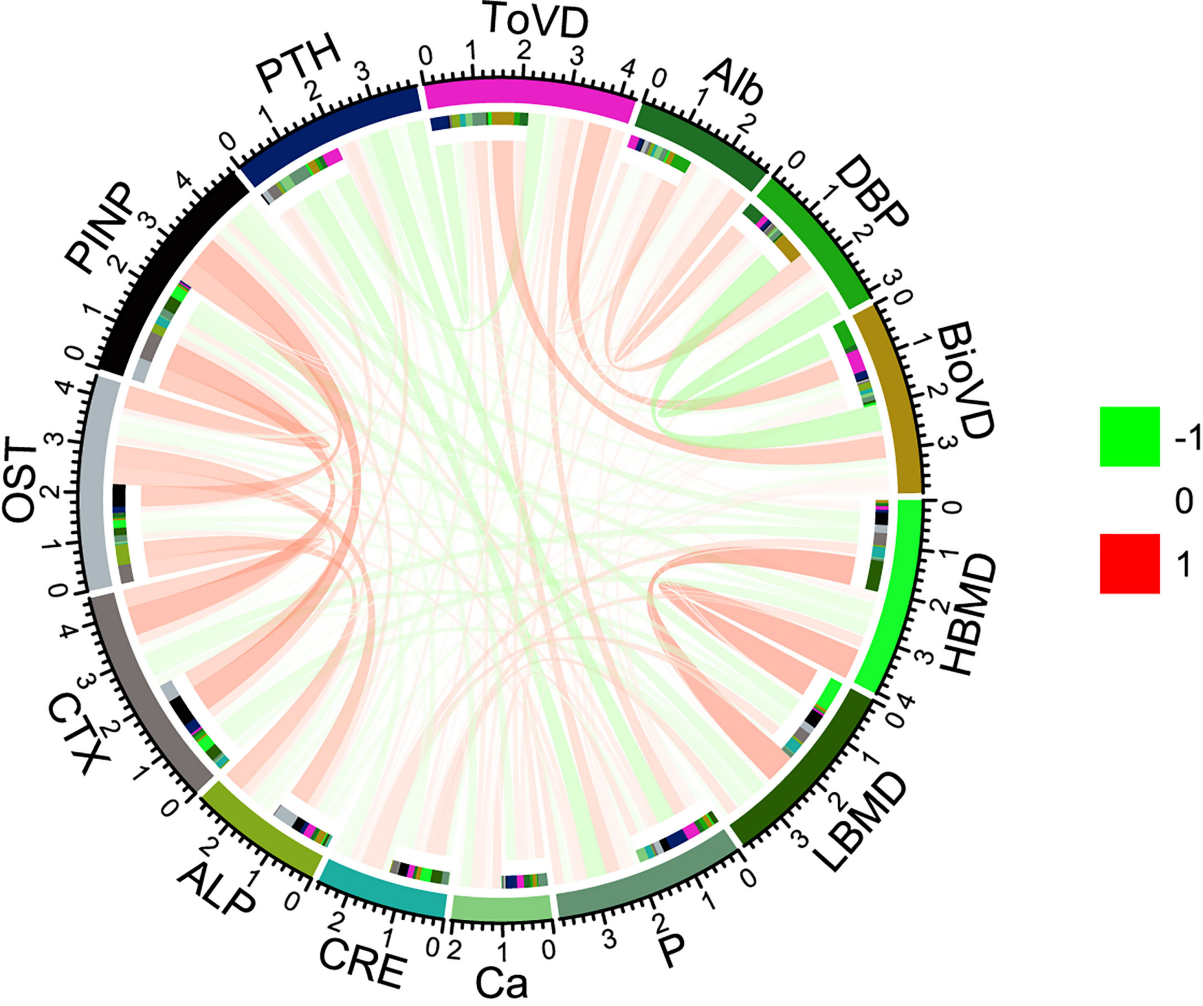
Spearman correlations among serum measures and BMD. The correlation coefficient between each pair of parameters is shown with a specific color and width of the line. Red lines represent positive correlations and green lines negative correlations. HBMD, Hip Bone Mineral Density; LBMD, lumbar bone mineral density; P, Phosphorous; Ca, Calcium; CRE, Creatinine; ALP, alkaline phosphatase; CTX, β-Cross Laps of type I collagen containing crosslinked C-telopeptide; OST, osteocalcin; PINP, N-terminal propeptide of type I procollagen; PTH, parathyroid hormone; ToVD, Total 25(OH)D; Alb, Albumin; DBP, vitamin D binding protein; BioVD, bioavailable 25(OH)D.

ToVD levels were positively correlated with BioVD, DBP and BMD levels and negatively correlated with the levels of the bone formation markers ALP and CTX, whereas BioVD levels did not correlate with the levels of any bone metabolism marker. All the bone metabolism markers demonstrated significant correlations with each other (p < 0.001, respectively). Ca and P showed statistical correlations with bone metabolism markers including OST, PINP and vitamin D status markers (including Alb, DBP and BioVD). It is interesting to note that CRE levels were positively correlated with the levels of the bone formation markers ALP, OST and PINP (p < 0.05, p < 0.001, p < 0.001, respectively), whereas they were not correlated with CTX levels statistically. Hip BMD may exhibit a high correlation with ToVD and PTH levels compared with that noted for lumbar BMD (**Figure 2**, **Tables S7**, **S8** in the **Supplementary Materials**).

**Figure 3:**
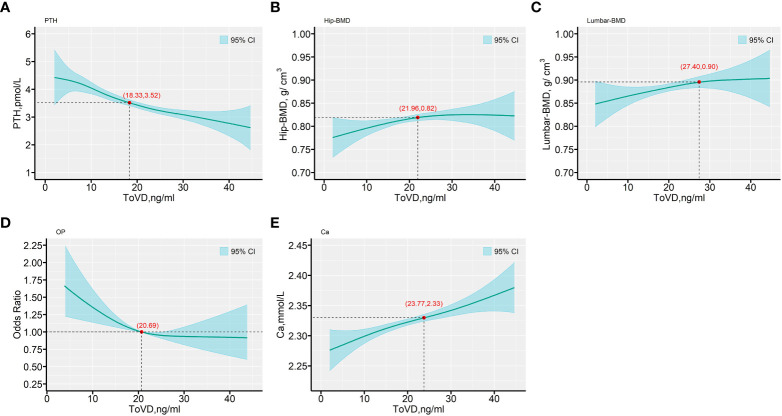
The predictive values for the outcomes of PTH, BMD biomarkers, OP risk and Ca, as functions of ToVD (ng/ml). **(A)** PTH; **(B)** Hip-BMD; **(C)** Lumbar-BMD; **(D)** OP; **(E)** Ca. The fitted values are shown in cyan lines, with a reference line provided (—). The Bayesian 95% confidence intervals are included in with cyan-color areas and calculated as described in the text. All plots are from generalized varying coefficient models (GVCM) with penalized cubic regression splines for the smooth coefficient functions. The smoothing parameters were selected by the generalized cross-validation (GCV) criterion. The red points with axis values denote the tangent points with the maximum changes of the predicted values by ToVD. In all panels, multivariate analyses were adjusted for age (in years, continuous), sex (men or women), educational status, smoking status, drinking status, dietary types, family income levels, seasons, regions and MAHS (in hours, continuous). The missing data were imputed by multiple imputation and all estimations were combined across the 5 imputed datasets using the Rubin’s method. ToVD, total 25(OH)D; PTH, parathyroid hormone; Hip-BMD, hip bone mineral density; Lumbar-BMD, lumbar bone mineral density; OP, osteoporosis; OR, odds ratio; Ca, Calcium; MAHS, mean annual hours sunshine; BMI, body mass index.

ToVD levels exhibited negative correlation with PTH levels (Spearman’s *r* = -0.384, p < 0.001). In addition, BioVD levels also exhibited negative correlation with PTH levels (Spearman’s *r* = -0.165, p < 0.05) (**Figure 2**, **Tables S7**, **S8** in the **Supplementary Materials**). According to the international guidelines, the vitamin D-sufficient threshold was mainly determined based on PTH and related markers, which were reassessed in the current study and presented in **Figure 3** and **Figure S4** in the **Supplementary Materials**. The adjusted generalized additive model exhibited a cutoff value of ToVD = 18.33 ng/ml for PTH, although the change was not apparent. The hip BMD exhibited a steep increase at ToVD < 21.96 ng/ml and a stationary phase at ToVD > 21.96 ng/ml. The same trend was found in the plot of the lumbar BMD with the exception that the cutoff of ToVD was 27.40 ng/ml. The analysis of the risk of developing osteoporosis demonstrated that when ToVD was less than 20.69 ng/ml, the odds ratio of osteoporosis was steadily increased. Ca levels exhibited a cutoff at ToVD = 23.77 ng/ml and were slowly increased. Unadjusted models are noted in **Figure S4** in the **Supplementary Materials** and all cutoff points of ToVD were less than 30 ng/ml.

### Interactive effects of 25(OH)D levels and BMI on BMD

**Figure 4:**
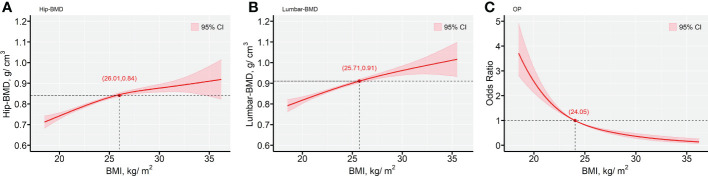
The predictive values for the outcomes of BMD biomarkers and OP risk, as functions of BMI (kg/m^2^). **(A)** Hip-BMD; **(B)** Lumbar-BMD; **(C)** OP. The fitted values are shown in red lines, with a reference line provided (—). The Bayesian 95% confidence intervals are included in with red-color areas, calculated as described in the text. All plots are from generalized varying coefficient models (GVCM) with penalized cubic regression splines for the smooth coefficient functions. Smoothing parameters were selected by the generalized cross-validation (GCV) criterion. The red points with axis values denoted the tangent points with the maximum changes of the predicted values by BMI. In all panels, multivariate analyses were adjusted for age (in years, continuous), sex (men or women), educational status, smoking status, drinking status, dietary types, family income levels, seasons, regions and MAHS (in hours, continuous). The missing data were imputed by multiple imputation and all estimations were combined across the 5 imputed datasets using the Rubin’s method. Hip-BMD, hip bone mineral density; Lumbar-BMD, lumbar bone mineral density; OP, osteoporosis; OR, odds ratio; MAHS, mean annual hours sunshine; BMI, body mass index.

**Figure 4** indicated the nonlinear modification by BMI for the effects of the predicted values of Hip-BMD, Lumbar-BMD and OP risk. The plots indicated the predicted values of Hip-BMD, which were positively associated with the function indicating increased BMI exposure, notably for the subjects with BMI > 26.01 kg/m^2^. Similar results were also observed with regard to the association between Lumbar-BMD and BMI, notably for the subjects with BMI > 25.71 kg/m^2^. However, the increasing BMI value was associated with the decrease in the OP risk that was demonstrated in the predicted risk curve in the GVCM, notably for the subjects with BMI > 24.05 kg/m^2^. The unadjusted results indicated similar results (**Figure S5** in the **Supplementary Materials**).

The results of the interactions between the covariate ToVD and the effect modifier BMI for Hip-BMD, Lumbar-BMD and OP risk outcomes are illustrated in **Figure S6** in the **Supplementary Materials**. The slope values of ToVD indicated a positive association of ToVD by BMI for Hip-BMD and Lumbar-BMD outcomes. In addition, a constant non-zero slope line for ToVD modified by BMI demonstrated a synergistic effect for the Lumbar-BMD outcome. The interaction between ToVD and BMI with regard to the OP risk outcome was negative, however it was not significant considering that the 95% CIs of the slope values were approaching zero. The unadjusted results indicated similar result in **Figure S7** of the **Supplementary Materials**.

**Figure 5:**
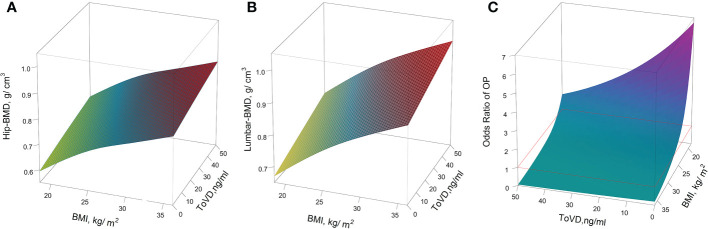
The predictive values for the outcomes of BMD biomarkers and OP risk, as functions of ToVD (ng/ml) and BMI (kg/m^2^). **(A)** Hip-BMD; **(B)** Lumbar-BMD; **(C)** OP. The yellow-colored region maps indicate the lower levels of the BMD area, and the red-colored area corresponds to the higher level of the BMD area; In the plot of the expression levels of Lumbar-BMD according to ToVD levels and BMI, The yellow-colored region maps the lower level of the BMD area, whereas the red-colored area corresponds to the higher level of the BMD area; In the plot of OP risk trajectory according to ToVD levels and BMI, compared with the reference values, the cyan-colored region maps the lower risk area and the red violet colored area corresponds to the higher risk area. Red lines are used to illustrate reference risk levels (*OR*=1, subjects with BMI = 24 kg/m^2^ and ToVD = 20.88 ng/ml as reference). In all panels, multivariate analyses were adjusted for age (in years, continuous), sex (men or women), educational status, smoking status, drinking status, dietary types, family income levels, seasons, regions and MAHS (in hours, continuous). The missing data were imputed by multiple imputation and all estimations were combined across the 5 imputed datasets using the Rubin’s method. ToVD, total 25(OH)D; Hip-BMD, hip bone mineral density; Lumbar-BMD, lumbar bone mineral density; OP, osteoporosis; OR, odds ratio; MAHS, mean annual hours sunshine; BMI, body mass index.

In the three-dimensional illustration of the trajectory of the level of Hip-BMD, Lumbar-BMD and OP risk were adjusted by covariates according to ToVD levels and BMI, respectively (**Figure 5**). The unadjusted results are indicated in **Figure S8** in the **Supplementary Materials**. The levels of BMD were increased with the BMI and ToVD levels and with their interaction (p < 0.001). The peak of BMD was observed among obesity subjects (BMI > 30 kg/m^2^) with the concentration of 40 to 50 ng/ml of ToVD (**Figures 5A, B**). In the three-dimensional illustration of the OP risk, it was found that the lower levels of ToVD and BMI would increase the risk of OP statistically, notably for those subjects with lower ToVD (less than 20.69 ng/ml) combined with lower BMI (less than 24.05 kg/m^2^) (**Figure 5C**).

## Discussion

25(OH)D levels are consistently lower in obese than in normal and overweight subjects. Therefore, obese individuals are frequently classified as VD deficient (22, 23). The present study involved middle-elder community-dwelling adults throughout China and the data indicated that lower 25(OH)D levels and higher BMD were confirmed for obese or high BMI subjects. We investigated the concrete additive effects of lower 25(OH)D levels and higher BMI on bone health among a large healthy sample size in China. The data indicated that higher BMI may have a beneficial effect on BMD, overshadowing the impact of reduced 25(OH)D levels, further the beneficial cutoff values of BMI and VD were given to maintain bone health, which could effectively reduce the burden of osteoporosis and its related fractures.

Reduced 25(OH)D levels are often accompanied with low BMD levels (14, 24). However, subjects with higher BMI and lower 25(OH)D levels exhibited higher BMD in the present study. This could be caused by the different levels of the bioavailable 25(OH)D. Bioavailable 25(OH)D consists primarily of albumin binding 25(OH)D (10% to 15% total 25(OH)D) and its free form (less than 10% of total 25(OH)D), acting directly on target tissue and cells (25). It could have a stronger effect on BMD compared with total 25(OH)D levels, as determined in our previous study (26). Therefore, the current study examined the difference of bioavailable 25(OH)D and its percentage in 25(OH)D among the three different BMI groups. The results indicated no significant differences among the three groups. Subsequently, the differences of the related metabolic indicators were examined among the different BMI groups. The participants of the obese group exhibited higher PTH levels compared with those of the normal group. PTH is a center hormone regulating calcium and phosphate levels through its actions at several organs. Its biological function resembles 1α, 25(OH)_2_D in increasing serum calcium. A higher PTH would in turn inhibit the production and secretion of 25(OH)D (27), a precursor of 1α, 25(OH)_2_D, resulting in BMD reduction. The characteristics of bone metabolism of the subjects investigated in the current study demonstrated that obese participants exhibited reduced concentration levels of OST and PINP. Concomitantly, these subjects experienced lower CTX levels, which may not weaken the influence of OST and PINP on BMD levels. The aforementioned finding has been previously confirmed in obese children and adolescents (28).

Genetic evidence pointing to lower VD and higher BMD is necessary. According to the genome-wide association study (GWAS) data (29) and a previous study published by our group (26), we genotyped rs12785878, rs10741657, rs4588, rs7041, rs2282679 and rs6013897, the VD related metabolism and transportation proteins gene locus SNPs information, finding absence of SNPs distribution differences among the three groups with the exception of the haplotypes. The Gc2 variant (TT for rs7041 and TT rs4588) accounted for the majority of obese homozygous participants. Due to the lower affinity constant between 25(OH)D and DBP, subjects carrying Gc2 always acquired lower 25(OH)D (21), which may explain why the obese group exhibited lower 25(OH)D levels. A research study on the association between DBP SNPs and obesity (30) revealed that certain DBP SNPs were significantly correlated with percentages of human fat mass. Therefore, the potential of the sampling to increase the susceptibility of obese subjects to carry Gc2 requires detailed investigation and verification in a larger sample size. No statistical associations were observed between the expression of specific genes and BMD in order to explain the higher BMD value noted in obese subjects. In addition, codominant, dominant and recessive models were applied to identify the susceptible SNPs in the six gene loci related to osteoporosis. The findings indicated that the gene locus SNPs included in the present study exhibited no association with the risk of developing osteoporosis.

In addition to the genetic factors, stores of 25(OH)D and serum25(OH)D are limited in humans (23). As a result, obese subjects would experience higher volume of distribution of 25(OH)D and bioavailable 25(OH)D. Sunlight exposure plays a key role in 25(OH)D_3_ production, which is a main component for the production of 25(OH)D. Reduced exposure to sunlight occurs in obese subjects, due to their reduced outdoor activities, which would also explain the diversity of 25(OH)D levels between obese and normal-weight subjects (31, 32). Given that overconsumption of food always leads to obesity, subjects with obesity often suffer from micronutrient deficiencies, notably hypovitaminosis D (33, 34). In contrast to these findings, adipose tissue acts as the most important storing compartment of VD and its analogues (35), which may contribute to the reduced VD levels in the obese population.

Although 25(OH)D levels were reduced in obese subjects, they cannot be used for pathological diagnosis. According to the guidelines on the management of VD deficiency in adults published in 2013 by the United Kingdom National Osteoporosis Society (36), the deficiency in serum 25(OH)D levels suggests that the serum concentration of 25(OH)D is below 30 ng/ml. Based on this threshold, the majority of the participants (nearly 80% of total participants) would be diagnosed as VD deficient. By comprehensively considering the osteoporosis occurrence risk, PTH reduction level and other indicators, the range from 18.33 to 27.40 ng/ml may be an optimization for the VD threshold, which again supports the conclusion that the VD deficiency threshold should be assessed according to pathological situations and ethnicity rather than adhering to a single standard.

Obesity or high BMI is recognized as a disease related to physical and psychological disorders (37). This may be an exception for BMD. High BMI is beneficial to lumbar or hip BMD and excessively higher BMI will not proportionally contribute to BMD accumulation. Reduced exposure to sunlight, decreased will to exercise and the negative influence of adipose tissue mass on bone caused by elevated BMI may counteract the advantage of weight-bearing effects (38). The participants of the present study exhibited a BMI ranging from 24.05 to 26.01 kg/m^2^. This can provide benefits to BMD and osteoporosis prevention. In other words, from the perspective of bone health, this range is suitable for Chinese elderly subjects, which is consistent with previously reported findings (39–41).

Interactive effects of lower 25(OH)D and higher BMI on BMD and osteoporosis were explored by constructing statistical models. We found that BMI and 25(OH)D always exerted interactions with BMD. The consideration that the BMI or 25(OH)D effect occurs separately on BMD is not suitable in Chinese elderly subjects. Those with lower 25(OH)D levels (< 20.69 ng/ml) combined with lower BMI (< 24.05 kg/m^2^) would suffer from higher risk of osteoporosis. Up to now, only a limited number of studies (23, 42) examined the associations among BMD, BMI and 25(OH)D levels, without comprehensive considerations for each of these parameters. Our study confirms a complex and bidirectional regulation of BMI and 25(OH)D for BMD, which deserves further exploration in the future.

The current study contains certain limitations which will be important to consider in future studies. Firstly, the current findings indicated a complex, nonlinear relationship between ToVD levels and the outcomes of BMD and OP risk modified by BMI status that may advance existing research examining these potential correlations among different outcomes. It is important to note that statistical associations do not imply causal relationships. However, our findings provide the biological plausibility of associations for future studies when evaluating these types of associations. Secondly, due to the cross-sectional design of our study, possible information and selection bias as well as other confounding variables, unknown or not were not considered in our statistical analysis. Therefore these factors may have influenced the reported results. Finally, we applied multiple statistical tests in our study, which increases the chance of type I errors. In the present study, a Bonferroni’s correction was applied to control the family-wise error rate.

## Conclusion

In conclusion, for middle-elder obese people, higher BMI may compensate for the adverse effects of lower 25(OH)D levels on BMD. Since 25(OH)D levels interact with BMI, BMI will most likely need to be considered when 25(OH)D levels are used to evaluate bone health. By retaining BMI at approximately 24.05 kg/m^2^ and VD at approximately 20.69 ng/ml, several benefits can be provided for Chinese elderly subjects.

## Data availability statement

The raw data supporting the conclusions of this article will be made available by the authors, without undue reservation.

## Ethics statement

The studies involving human participants were reviewed and approved by Longhua Hospital affiliated to the Shanghai University of Traditional Chinese Medicine. The patients/participants provided their written informed consent to participate in this study.

## Author contributions

YW and DT had full access to the data in the study and take responsibility for the integrity of the data and the accuracy of the data analysis. CY, JW, WZ, HY, and BS are co-firster authors. YW and DT are co-corresponding authors. Concept and design, CY, WZ, DT, and YW. Acquisition, analysis, or interpretation of data, all authors. Drafting of the manuscript, WZ, CY, and HY. Critical revision of the manuscript for important intellectual content, DT, YW, WZ, CY, JW, and BS. Statistical analysis, HY, CY, and WZ. Obtained funding, YW, BS, QL, HW, PC, and DT. Administrative, technical, or material support, CY, JW, WZ, HY, BS, CL, QL, DL, BC, XX, XL, XW, HW, PC, CH, HX, YS, YZ, QS, DT, and YW. Supervision, DT, CY, WZ, HY, BS, JW, and YW. All authors contributed to the article and approved the submitted version.
