# Supplementary material for: Effects of obesity with reduced 25(OH)D levels on bone health in elderly Chinese people: a nationwide cross-sectional study
Source: Front Immunol. 2023 Apr 27;14:1162175. doi: 10.3389/fimmu.2023.1162175 (PMC10172581; doi:10.3389/fimmu.2023.1162175)
Supplement: Supplementary file 1 [file DataSheet_1.docx]

**Supplementary Materials**

**Supplementary Methods**

**Table S1.** Laboratory detection methods

**Table S2.** The concerned gene locus SNP information in the study

**Table S3.** Adjusted p value of BMD and ToVD among groups

**Table S4.**  Comparision of serum biomarkers and physical measurements among different BMI participants without imputation

**Table S5.** Association of haplotype, ToVD and Hip-BMD

**Table S6.** Genetic difference in normal, overweight and obese group

**Table S7.** The p values of the correlations between markers of vitamin D status and bone metabolism markers

**Table S8.**  The correlation coefficients between markers of vitamin D status and bone metabolism markers

**Figure S1.** Participant recruitment and group

**Figure S2.**  Heatmap of genetic data

**Figure S3.** Proportion of haplotype among normal, overweight and obese group

**Figure S4.** The predictive values for the outcomes of PTH, Hip-BMD, Lumbar-BMD, OP risk and Ca analyzed, as functions of ToVD (ng/ml) in the unadjusted model

**Figure S5.** The predictive values for the outcomes of PTH, Hip-BMD, Lumbar-BMD, OP risk and Ca analyzed, as functions of BMI (kg/m^2^) in the unadjusted model

**Figure S6.** Varying coefficient functions of the ToVD slope values, for the Hip-BMD, Lumbar-BMD and OP risk outcomes analyzed, as functions of BMI (kg/m^2^).

**Figure S7.** Varying coefficient functions of the ToVD slope values, for the Hip-BMD, Lumbar-BMD and OP risk outcomes analyzed, as functions of BMI (kg/m^2^) in the unadjusted model

**Figure S8.**  Three-Dimensional Illustrations of Hip-BMD, Lumbar-BMD, and OP risk according to ToVD levels and BMI in the unadjusted model

**Supplementary References**

**Supplementary Methods**

***Genotyping***

The DNA samples were genotyped for the presence of six single nucleotide polymorphisms (SNPs) (rs12785878, rs10741657, rs4588, rs7041, rs2282679 and rs6013897) related to vitamin D production, metabolism and transportation with the TaqmanSNP genotyping assays (Thermo Fisher Scientific, USA). These polymorphisms were selected based on the following evidence:1) rs12785878, rs10741657, rs6013897 and rs2282679 were identified to be accountable for 5% of the 25(OH)D variability^1,2^ and 2)rs4588 and rs7041 were significantly associated with the 25(OH)D concentration and with BMD levels as reported in our previous study ^3^and other studies^4,5^.

***Generalized varying coefficient model***

Since the relationship between outcomes and explanatory covariates may not necessarily be linear or polynomial, generalized varying coefficient models (GVCM) were employed in this study. The GCVM is a regression model, which is additive in the regressors, but no longer assumes a linear effect of the regressors. In contrast to the generalized linear models (GLM) with fixed coefficients for each explanatory covariate, a smooth function of an effect modifier was used to present the relationship between an explanatory covariate and the outcome changing at different levels of the effect modifier. The functions can either be flexible parametric functions, or more generally non-parametric functions.

The GVCM with a single effect modifier has the following general form:

$$g(\mu)=b_{0}+\beta_{0}\left( M \right)+\sum_{i=1}^{K} \beta_{i}\left( M \right)\cdot X_{i}+\sum_{j=1}^{s} b_{j}Z_{j}+\varepsilon$$

Where g($\mu$) is a link function as in GLMs. *Y* is a normally distributed outcome variable with mean$\mu$, g($\mu$) = E(*Y*) =$\mu$. *Y* is a binary variable and the model is a logistic model with g($\mu$) = $log(\frac{\mu}{1-\mu})$. *M* is the single effect modifying the explanatory covariate. *b*_0_ is the constant intercept and *β*_0_(*M*) is the varying intercept representing a main effect of *M*, which is modeled as a smoothly varying function of *M*. *X_i_* are explanatory covariates, *β_i_*(M) is the potentially nonlinear tensor product based smooth of *M*, for the *i* = 1,2, …, *K* other explanatory variables, which reflects the relationship between covariate *X_i_* and outcome changes as the levels of the effect modifier change. *K* is the number of internal knots of the smoothing parameter estimation in the spline function, which were selected by the generalized cross-validation (GCV) criterion. Therefore, the relationship between the explanatory variables *X_i_* and *M* characterizes a type of 2-way interactions between those two covariates. *Z_j_* are additional covariates with fixed coefficients *b_j_*, where *j* = 1,2,…,*s*. *ε* is an error term.

The analysis in the present study used non-parametric techniques to identify a GVCM with penalized cubic regression splines for the smooth coefficient functions. The linearity or nonlinearity of the smoothed functions were assessed by a likelihood ratio test which asymptotically followed a chi-square distribution. In the penalized regression spline estimation, a penalty was used to regulate the smoothness of the spline following placement of a sufficiently large number of knots. The gam.check function in the ‘*mgcv*’ R package was also used to assess whether the knots were appropriately set. The figures were created to present estimated effects with Bayesian 95% confidence intervals (CI) and the association of *X_i_* with the outcome, as a function of the level of M. The predicted outcomes or odds ratios with 95% confidence intervals (CIs) were used depending on the type of outcomes.

***Generalized varying coefficient model with R***

Generalized varying coefficient models (GVCM) with penalized cubic regression spline functions were estimated using the *mgcv* package in R software and using the *gam* function with the “by” option, and of the number of internal knots of the smoothing parameter estimation were chosen by the generalized cross-validation (GCV) criterion. Selection of the penalty parameter in the penalized spline representation can be accomplished automatically.

The R codes were shown in the following example:

gam(hipBMD ~ te(BMI, bs = "cr") + te(ToVD, bs = "cr") + te(BMI,by=ToVD) + sex

where hipBMD is the response variable for BMD, sex is the independent variable with constant coefficients, bs is for a penalized cubic regression spline smooth which have a cubic spline basis defined by a modest sized set of knots spread evenly through the covariate values, and the penalty modified to shrink towards zero at high enough smoothing parameters.

The effective degree of freedom (EDF) indicator was used for determining a non-linear relationship between the covariates and the outcome variable. If the EDF value great than 1 or less than 1 means that the smooth function is nonlinear, otherwise it is linear. So the EDF values of the smooth term derived from GVCM characterize the shape (linear or non-linear) of the smooth function.

For plots produced by the *plot.gam* function of the *mgcv* package, Bayesian confidence intervals are used for plotting of the smooth terms. For model selection, testing between nested models was performed using anova(model_1,model_2, test="Chisq"). In addition the AIC of the models were found using the AIC function in R.

**Table S1. Laboratory detection methods**

| **Index** | **Methods** | **Detecting instrument** |
| --- | --- | --- |
| **PTH** | Electrochemiluminescence | Automatic biochemical analyzer (cobas 8000 e602, Roche) |
| **Alb** | Bromocresol green (BCG) dye binding | Automatic biochemical analyser (modular P800, Roche) |
| **CRE** | Enzymatic | Roche Creatinine Plus assay |
| **Ca** | O-cresolphthalein complexone | Automatic biochemical analyser (modular P800, Roche) |
| **P** | O-cresolphthalein complexone | Automatic biochemical analyser (modular P800, Roche) |
| **ALP** | Continuous monitoring technique | Automatic biochemical analyzer (modular P800, Roche) |
| **PINP** | Electrochemiluminescence | Automatic biochemical analyzer (cobas 8000 e602, Roche) |
| **OST** | Electrochemiluminescence | Automatic biochemical analyzer (cobas 8000 e602, Roche) |
| **CTX** | Electrochemiluminescence | Automatic biochemical analyzer (cobas 8000 e602, Roche) |

Abbreviations: PTH, parathyroid hormone; Alb, Albumin; CRE, Creatinine; Ca, Calcium; P, Phosphorous; ALP, alkaline phosphatase; PINP, N-terminal propeptide of type I procollagen; OST, osteocalcin; CTX, β-CrossLaps of type I collagen containing crosslinked C-telopeptide;

**Table S2.** **The concerned gene locus SNP information in the study**

| **Gene names** | **Enzyme/protein** | **Locus** | **SNP types** |
| --- | --- | --- | --- |
| DHCR7/NADSYN1 | 7-dehydrocholesterol reductase | rs12785878 | T/T, T/G, G/G |
| CYP2R1 | 25-hydroxylase | rs10741657 | AA, A/G, G/G |
| CYP24A1 | 25-hydroxylase | rs6013897 | T/T, A/T, A/A |
| GC | DBP | rs4588 | T/T, T/G, G/G |
| GC | DBP | rs7041 | T/T, T/G, G/G |
| GC | DBP | rs2282679 | T/T, T/G, G/G |

Abbreviations: GC, Group specific component; DBP, vitamin D binding protein.

**Table S3. Adjusted p value of BMD and ToVD among groups**

|  | **Adjusted^b^** | | | |
| --- | --- | --- | --- | --- |
|  | *P* Value^a^ | 1 vs 2 | 1 vs 3 | 2 vs 3 |
| Hip-BMD (g/cm^3^) | ***<0.001*** | ***<0.001*** | ***<0.001*** | ***<0.001*** |
| Lumbar-BMD (g/cm^3^) | ***<0.001*** | ***<0.001*** | ***<0.001*** | ***<0.001*** |
| ToVD (ng/ml) | ***0.040*** | 1.000 | ***0.047*** | 0.243 |

Abbreviations: 1: Normal group; 2: Overweight group; 3: Obese group. BMD, Bone Mineral Density, ToVD, Total 25(OH)D.

^a^ p values were estimated by multiple linear regression models using multiple imputations (the number of imputations was 5), and adjusted coefficients and standard errors for the variability between imputations according to the combination rules by Rubin. The significant p values were highlighted by bold, italics font. The skewed dependents variables were transformed using the Box-Cox transformation to approach normality for the linear regression model. Bonferroni's adjustment for multiple comparisons were used for pairwise comparisons among groups after the multiple imputation estimations. The p values for pairwise comparisons were significant at the familywise error rate of 0.05 by Bonferroni's adjustment.

^b^ Adjusted by season, region, sex, age, education, salary, smoking, drinking, diet and mean annual hours sunshine.

**Table S4.** **Comparision of serum biomarkers and physical measurements among different BMI participants without imputation**

|  | **p value ^a^** | **p value ^b^** | **Adjusted p value ^c^** |
| --- | --- | --- | --- |
| Hip-BMD (g/cm^3^) | <0.001 | <0.001 | <0.001 |
| Lumbar-BMD (g/cm^3^) | <0.001 | <0.001 | <0.001 |
| ToVD (ng/ml) | <0.001 | <0.001 | 0.040 |
| PTH (pmol/L) | <0.001 | <0.001 | 0.030 |
| Alb (g/L) | 0.051 | 0.054 | 0.066 |
| BioVD (ng/ml) | 0.072 | 0.117 | 0.545 |
| Percentage (%)^d^ | 0.463 | 0.773 | 0.812 |
| CRE (μmol/L) | 0.115 | 0.825 | 0.314 |
| Ca (mmol/L) | 0.089 | 0.042 | 0.303 |
| P (mmol/L) | <0.001 | <0.001 | 0.582 |
| DBP (μg/mL) | 0.217 | 0.683 | 0.565 |
| ALP (U/L) | 0.1 | 0.001 | 0.387 |
| PINP (ng/ml) | <0.001 | <0.001 | 0.011 |
| OST (ng/ml) | <0.001 | <0.001 | <0.001 |
| CTX (ng/ml) | <0.001 | <0.001 | <0.001 |
| LGS (kg) | <0.001 | <0.001 | <0.001 |
| RGS (kg) | <0.001 | <0.001 | 0.007 |
| FTSST (s) | <0.001 | <0.001 | 0.006 |
| PA (MET-min/w) | 0.045 | 0.146 | 0.303 |

Abbreviations: BMD, Bone Mineral Density, ToVD, Total 25(OH)D; PTH, parathyroid hormone; Alb, Albumin; BioVD, bioavailable 25(OH)D; CRE, Creatinine; Ca, Calcium; P, phosphorous; DBP, vitamin D binding protein; ALP, alkaline phosphatase; PINP, N-terminal propeptide of type I procollagen; OST, osteocalcin; CTX, β-CrossLaps of type I collagen containing crosslinked C-telopeptide; LGS, left grip strength; RGS, right grip strength; FTSST, five-times-sit-to-stand test; PA, physical activity

^a^ p value was gotten from Kruskal-Wallis *H* test.

^b^ p value was gotten from univariate linear regression.

^c^ Adjusted p value was estimated by multiple regression analysis, model including season, region, sex, age, education, salary, smoking, drinking, diet and mean annual hours sunshine.

^d^Percentage= BioVD/ Total 25(OH)D🞨100%

**Table S5. Association of haplotype, ToVD and Hip-BMD**

| **Haplotype** | **ToVD (ng/ml)** | **p value** | **Adjusted p value ^a^** | **Hip-BMD(g/cm^3^)** | **p value** | **Adjusted p value ^a^** |
| --- | --- | --- | --- | --- | --- | --- |
| **GC1F** | 21.8(16.3, 26.8) |  |  | 0.890(0.711, 0.899) |  |  |
| **GC1S** | 23.3(16.5, 28.5) | <0.001 | <0.001 | 0.810(0.718, 0.891) | 0.954 | 0.709 |
| **GC2** | 18.05(13.10, 22.9) |  |  | 0.805(0.730, 0.895) |  |  |

Abbreviations: ToVD, Total 25(OH)D; BMD, Bone Mineral Density

^a^ Adjusted p value was estimated by multiple regression analysis, model including season, region, sex, age, education, salary, smoking, drinking, diet and mean annual hours sunshine.

**Table S6. Genetic difference in normal, overweight and obese group**

| **Genotype** | **p value** | **Allele** | **p value** | |
| --- | --- | --- | --- | --- |
| rs12785878 | 0.237 | rs12785878 | 0.081 | |
| rs10741657 | 0.488 | rs10741657 | 0.692 | |
| rs4588 | 0.031 | rs4588 | 0.009 | |
| rs7041 | 0.595 | rs7041 | 0.760 | |
| rs2282679 | 0.050 | rs2282679 | 0.012 | |
| rs6013897 | 0.917 | rs6013897 | 0.691 | |
| Haplotype | 0.018 |  | |  |

**Table S7.** **The P-values of the correlations between markers of vitamin D status and bone metabolism markers**

|  | HBMD | LBMD | P | Ca | CRE | ALP | CTX | OST | PINP | PTH | ToVD | Alb | DBP | BioVD |
| --- | --- | --- | --- | --- | --- | --- | --- | --- | --- | --- | --- | --- | --- | --- |
| HBMD |  | <0.001 | 0.038 | 0.010 | <0.001 | 0.368 | <0.001 | <0.001 | <0.001 | <0.001 | <0.001 | 0.007 | 0.330 | 0.813 |
| LBMD | <0.001 |  | <0.001 | 0.197 | <0.001 | 0.433 | <0.001 | <0.001 | <0.001 | 0.909 | 0.026 | 0.466 | 0.006 | 0.484 |
| P | 0.038 | <0.001 |  | <0.001 | <0.001 | 0.664 | <0.001 | <0.001 | <0.001 | <0.001 | <0.001 | <0.001 | <0.001 | <0.001 |
| Ca | 0.010 | 0.197 | <0.001 |  | 0.091 | 0.258 | 0.654 | <0.001 | 0.042 | <0.001 | <0.001 | <0.001 | 0.009 | 0.006 |
| CRE | <0.001 | <0.001 | <0.001 | 0.091 |  | 0.019 | 0.096 | <0.001 | 0.001 | <0.001 | 0.001 | 0.074 | 0.053 | 0.110 |
| ALP | 0.368 | 0.433 | 0.664 | 0.258 | 0.019 |  | <0.001 | <0.001 | <0.001 | 0.020 | <0.001 | <0.001 | 0.068 | 0.052 |
| CTX | <0.001 | <0.001 | <0.001 | 0.654 | 0.096 | <0.001 |  | <0.001 | <0.001 | <0.001 | 0.002 | 0.001 | <0.001 | 0.103 |
| OST | <0.001 | <0.001 | <0.001 | <0.001 | <0.001 | <0.001 | <0.001 |  | <0.001 | <0.001 | 0.445 | <0.001 | 0.014 | 0.151 |
| PINP | <0.001 | <0.001 | <0.001 | 0.042 | 0.001 | <0.001 | <0.001 | <0.001 |  | <0.001 | 0.061 | 0.828 | 0.253 | 0.751 |
| PTH | <0.001 | 0.909 | <0.001 | <0.001 | <0.001 | 0.020 | <0.001 | <0.001 | <0.001 |  | <0.001 | <0.001 | 0.007 | 0.004 |
| ToVD | <0.001 | 0.026 | <0.001 | <0.001 | 0.001 | <0.001 | 0.002 | 0.445 | 0.061 | <0.001 |  | <0.001 | <0.001 | <0.001 |
| Alb | 0.007 | 0.466 | <0.001 | <0.001 | 0.074 | <0.001 | 0.001 | <0.001 | 0.828 | <0.001 | <0.001 |  | <0.001 | 0.027 |
| DBP | 0.330 | 0.006 | <0.001 | 0.009 | 0.053 | 0.068 | <0.001 | 0.014 | 0.253 | 0.007 | <0.001 | <0.001 |  | <0.001 |
| BioVD | 0.813 | 0.484 | 0.001 | 0.006 | 0.110 | 0.052 | 0.103 | 0.151 | 0.751 | 0.004 | <0.001 | 0.027 | <0.001 |  |

Abbreviations: HBMD, Hip Bone Mineral Density; LBMD, Lumbar Bone Mineral Density; P, Phosphorous; Ca, Calcium; CRE, Creatinine; ALP, alkaline phosphatase; CTX, β-CrossLaps of type I collagen containing crosslinked C-telopeptide; OST, osteocalcin; PINP, N-terminal propeptide of type I procollagen; PTH, parathyroid hormone; ToVD, Total 25(OH)D; Alb, Albumin; DBP, vitamin D binding protein; BioVD, bioavailable 25(OH)D.

**Table S8. The correlation coefficients between markers of vitamin D status and bone metabolism markers**

|  | HBMD | LBMD | P | Ca | CRE | ALP | CTX | OST | PINP | PTH | ToVD | Alb | DBP | BioVD |
| --- | --- | --- | --- | --- | --- | --- | --- | --- | --- | --- | --- | --- | --- | --- |
| HBMD | 1.000 | 0.675 | -0.047 | -0.021 | 0.240 | -0.037 | -0.277 | -0.176 | -0.270 | -0.064 | 0.083 | 0.058 | 0.015 | 0.054 |
| LBMD | 0.675 | 1.000 | -0.104 | -0.015 | 0.243 | -0.057 | -0.247 | -0.165 | -0.315 | 0.003 | 0.045 | -0.007 | -0.039 | 0.053 |
| P | -0.047 | -0.104 | 1.000 | 0.207 | -0.146 | -0.024 | -0.056 | 0.142 | 0.164 | -0.423 | 0.304 | 0.110 | 0.108 | 0.100 |
| Ca | -0.021 | -0.015 | 0.207 | 1.000 | -0.013 | -0.012 | -0.027 | 0.040 | 0.020 | -0.234 | 0.153 | 0.137 | 0.059 | 0.080 |
| CRE | 0.240 | 0.243 | -0.146 | -0.013 | 1.000 | -0.025 | -0.143 | -0.018 | -0.189 | -0.021 | 0.126 | 0.063 | 0.006 | 0.088 |
| ALP | -0.037 | -0.057 | -0.024 | -0.012 | -0.025 | 1.000 | 0.026 | 0.461 | 0.202 | 0.093 | -0.180 | -0.084 | 0.025 | -0.150 |
| CTX | -0.277 | -0.247 | -0.056 | -0.027 | -0.143 | 0.026 | 1.000 | 0.397 | 0.624 | 0.227 | -0.045 | -0.060 | -0.090 | 0.032 |
| OST | -0.176 | -0.165 | 0.142 | 0.040 | -0.018 | 0.461 | 0.397 | 1.000 | 0.496 | 0.135 | -0.004 | -0.099 | -0.022 | -0.035 |
| PINP | -0.270 | -0.315 | 0.164 | 0.020 | -0.189 | 0.202 | 0.624 | 0.496 | 1.000 | 0.035 | -0.030 | 0.023 | 0.021 | -0.028 |
| PTH | -0.064 | 0.003 | -0.423 | -0.234 | -0.021 | 0.093 | 0.227 | 0.135 | 0.035 | 1.000 | -0.384 | -0.103 | -0.091 | -0.165 |
| ToVD | 0.083 | 0.045 | 0.304 | 0.153 | 0.126 | -0.180 | -0.045 | -0.004 | -0.030 | -0.384 | 1.000 | 0.188 | 0.126 | 0.492 |
| Alb | 0.058 | -0.007 | 0.110 | 0.137 | 0.063 | -0.084 | -0.060 | -0.099 | 0.023 | -0.103 | 0.188 | 1.000 | 0.390 | 0.115 |
| DBP | 0.015 | -0.039 | 0.108 | 0.059 | 0.006 | 0.025 | -0.090 | -0.022 | 0.021 | -0.091 | 0.126 | 0.390 | 1.000 | -0.579 |
| BioVD | 0.054 | 0.053 | 0.100 | 0.080 | 0.088 | -0.150 | 0.032 | -0.035 | -0.028 | -0.165 | 0.492 | 0.115 | -0.579 | 1.000 |

Abbreviations: HBMD, Hip Bone Mineral Density; LBMD, Lumbar Bone Mineral Density; P, Phosphorous; Ca, Calcium; CRE, Creatinine; ALP, alkaline phosphatase; CTX, β-CrossLaps of type I collagen containing crosslinked C-telopeptide; OST, osteocalcin; PINP, N-terminal propeptide of type I procollagen; PTH, parathyroid hormone; ToVD, Total 25(OH)D; Alb, Albumin; DBP, vitamin D binding protein; BioVD, bioavailable 25(OH)D.

**Figure S1. Participant recruitment and group**


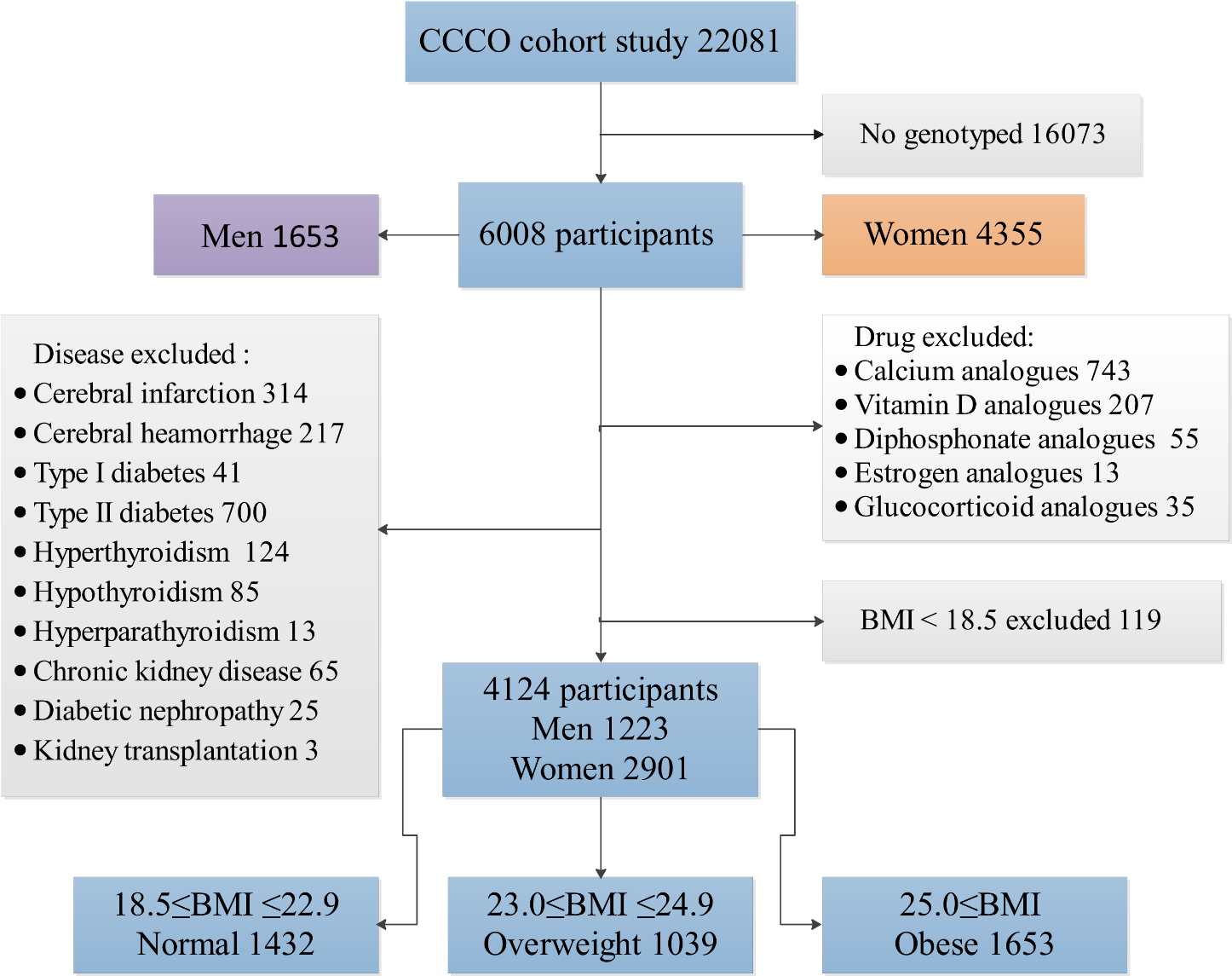


**Figure S2. Heatmap of genetic data**


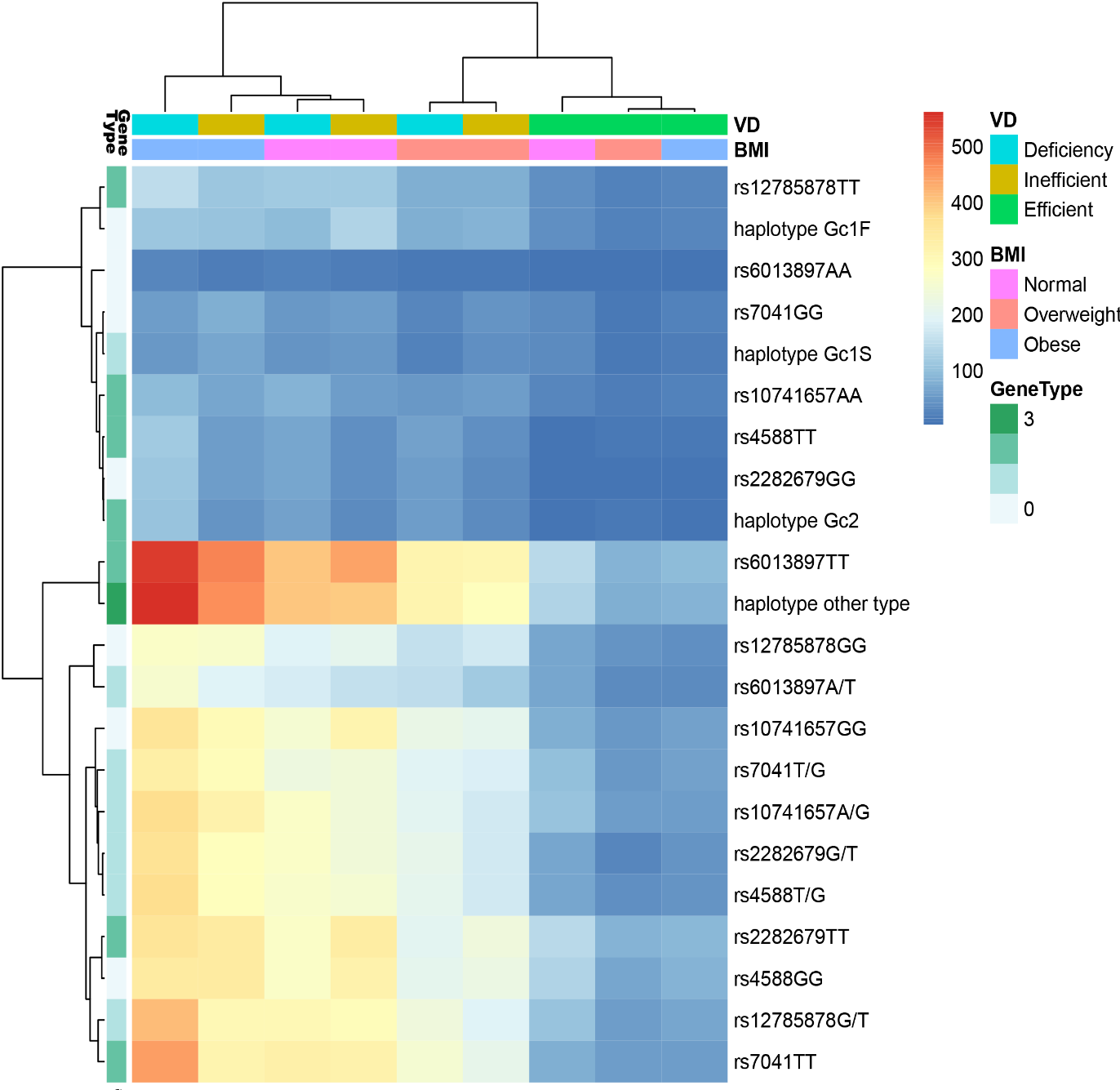


Abbreviations: VD, Total 25(OH)D; BMI, Body Mass Index.

**Figure S3. Proportion of haplotype among normal, overweight and obese group**


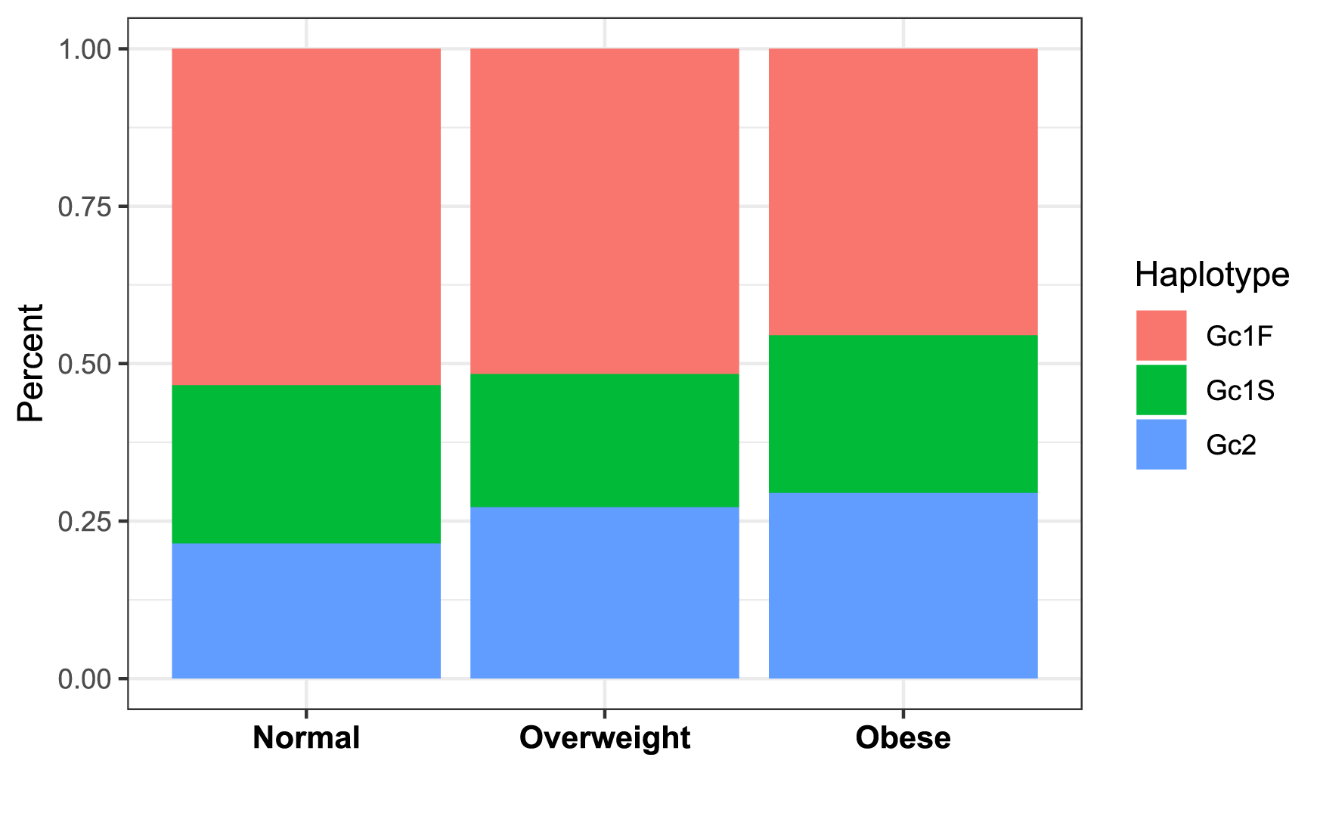


**Figure S4. The predictive values for the outcomes of PTH, Hip-BMD, Lumbar-BMD, OP risk and Ca analyzed, as functions of ToVD (ng/ml)** **in the unadjusted model**


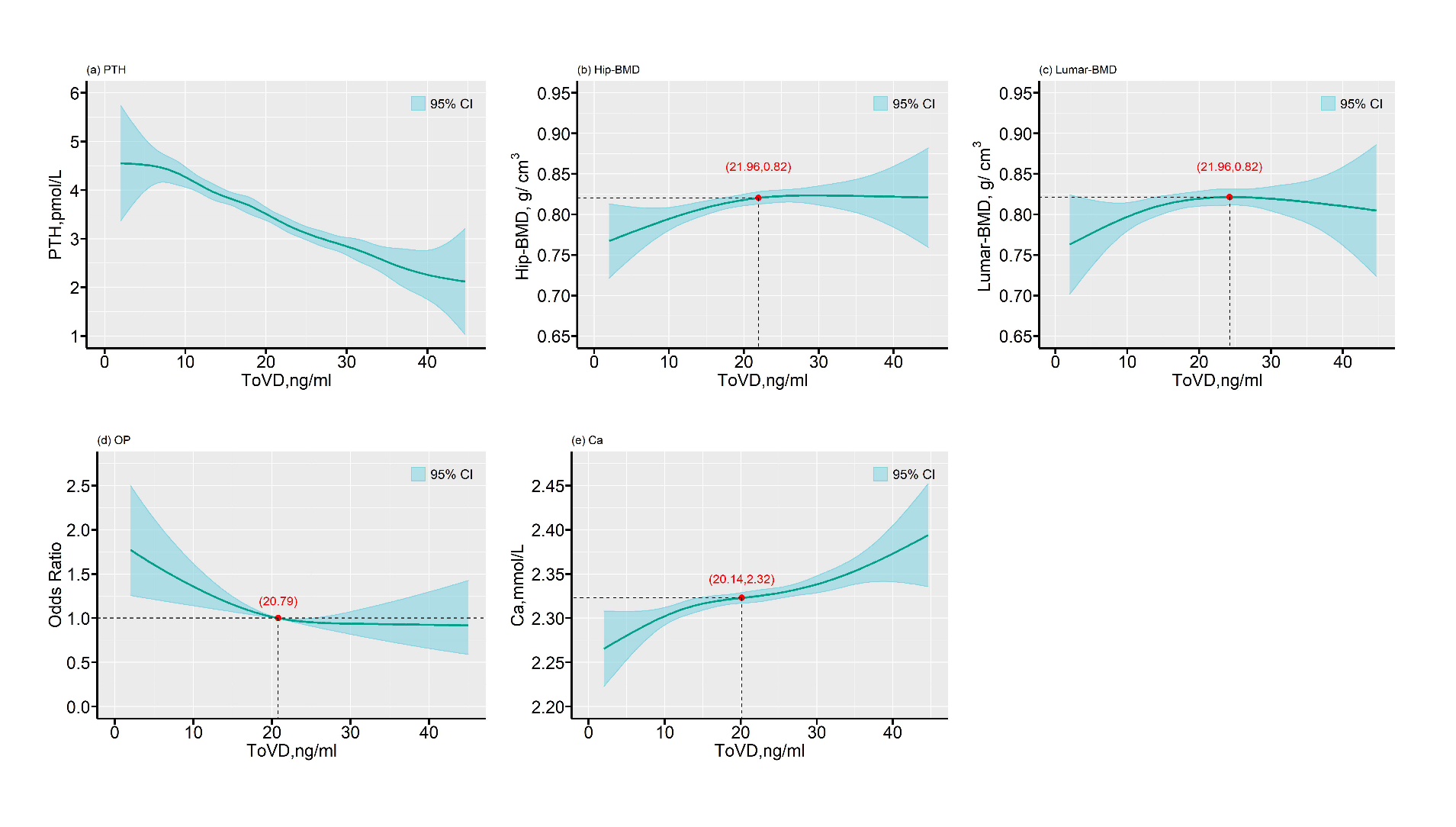


Abbreviations: ToVD, Total 25(OH)D; PTH, parathyroid hormone; Hip-BMD, hip bone mineral density; Lumbar-BMD, lumbar bone mineral density; OP, osteoporosis; Ca, Calcium.

**Figure S5.** **The predictive values for the outcomes of Hip-BMD, Lumbar-BMD, and OP risk analyzed, as functions of BMI (kg/m^2^) in the unadjusted model**


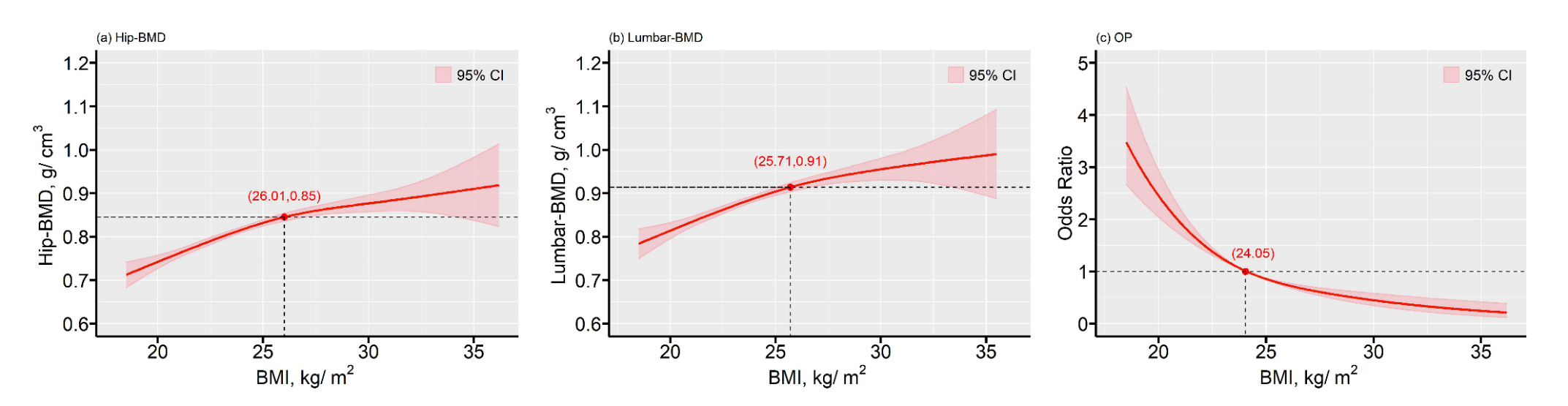


Abbreviations: BMI, Body Mass Index; Hip-BMD, hip bone mineral density; Lumbar-BMD, lumbar bone mineral density; OP, osteoporosis.

**Figure S6. Varying coefficient functions of the ToVD slope values, for the Hip-BMD, Lumbar-BMD and OP risk outcomes analyzed, as functions of BMI (kg/m^2^).**


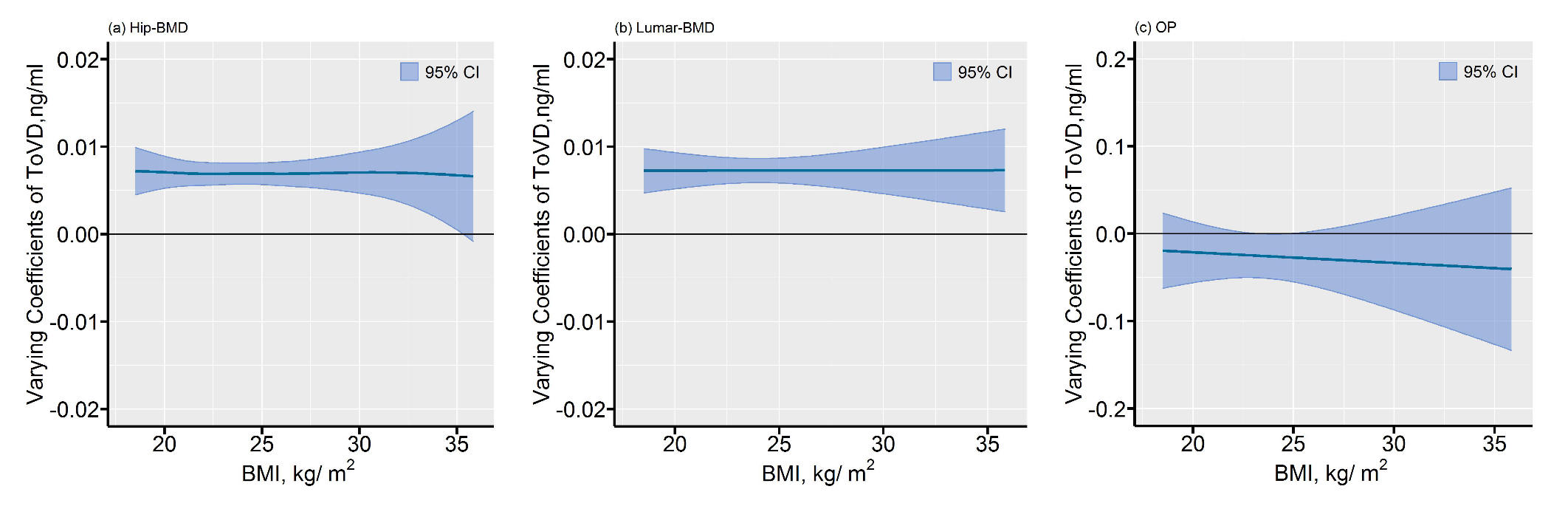


Abbreviations: BMI, Body Mass Index; ToVD, Total 25(OH)D; Hip-BMD, hip bone mineral density; Lumbar-BMD, lumbar bone mineral density; OP, osteoporosis.

**Figure S7. Varying coefficient functions of the ToVD slope values, for the Hip-BMD, Lumbar-BMD and OP risk outcomes analyzed, as functions of BMI (kg/m^2^) in the unadjusted model**


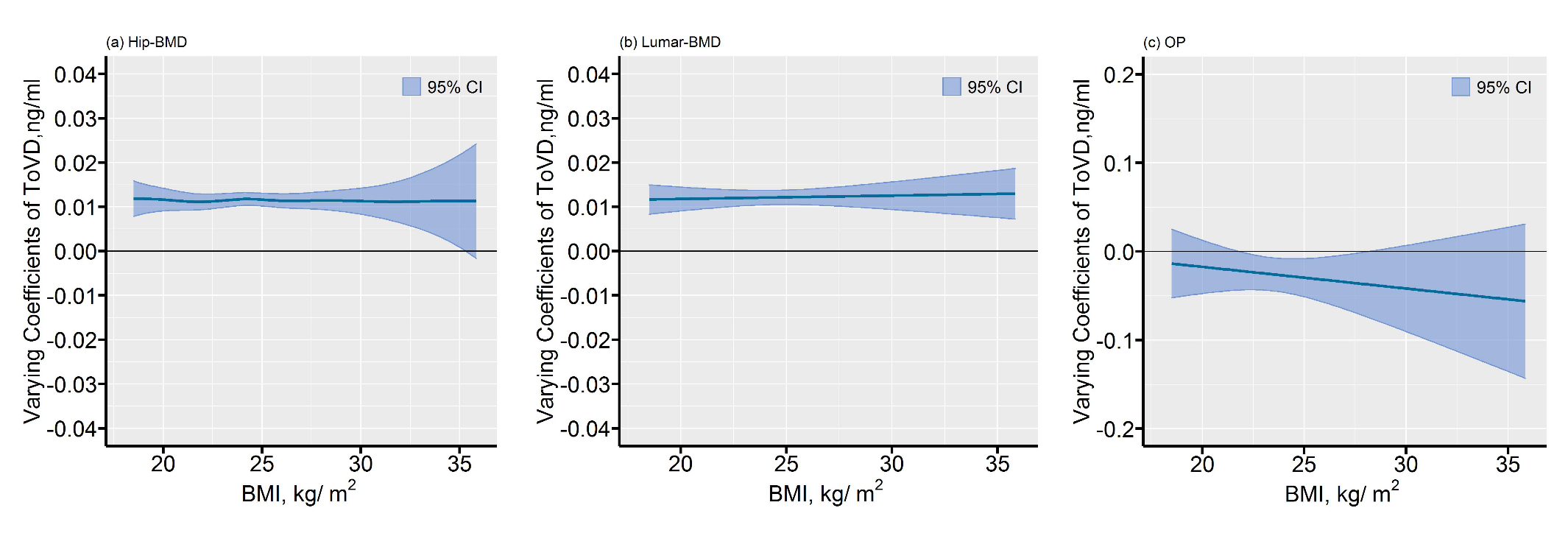


Abbreviations: BMI, Body Mass Index; ToVD, Total 25(OH)D; Hip-BMD, hip bone mineral density; Lumbar-BMD, lumbar bone mineral density; OP, osteoporosis.

**Figure S8.** **Three-Dimensional Illustrations of Hip-BMD, Lumbar-BMD, and OP Risk according to ToVD levels and BMI in the unadjusted model**


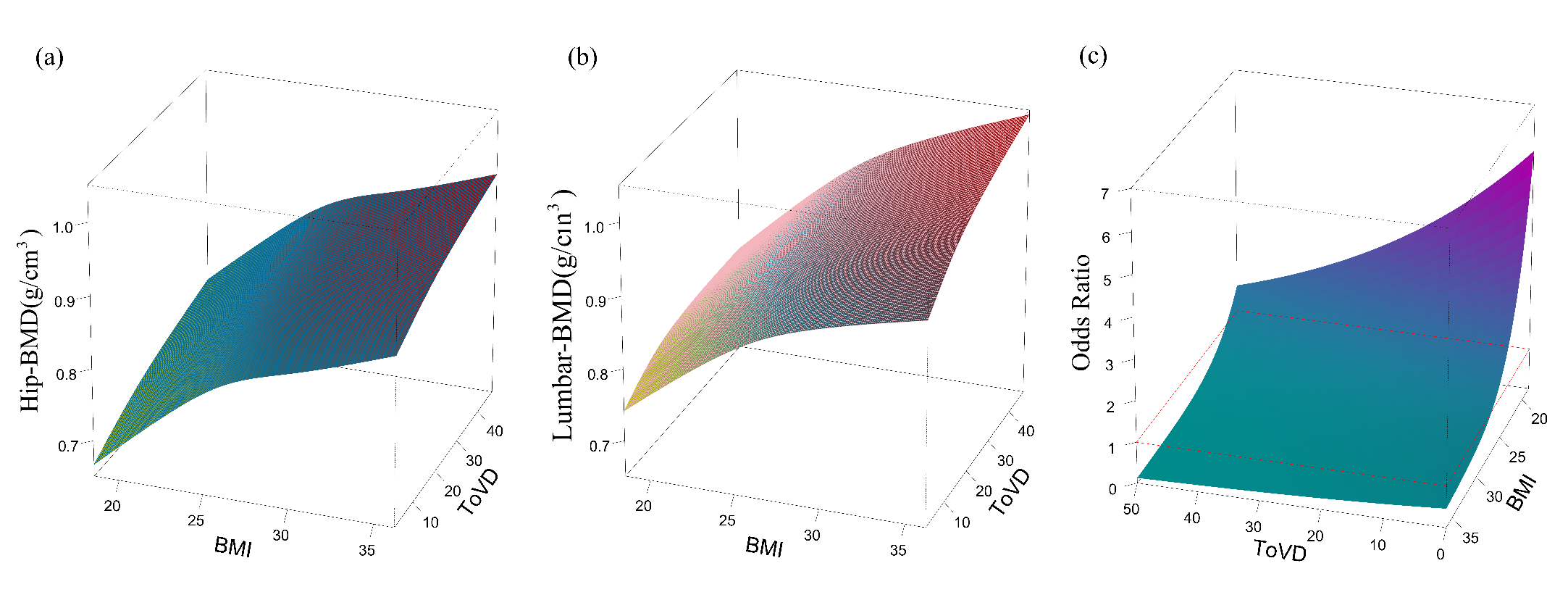


Abbreviations: BMI, Body Mass Index; ToVD, Total 25(OH)D; Hip-BMD, hip bone mineral density; Lumbar-BMD, lumbar bone mineral density; OP, osteoporosis.

**Supplementary References**

1. Adami S, Bertoldo F, Braga V, et al. 25-hydroxy vitamin D levels in healthy premenopausal women: association with bone turnover markers and bone mineral density. Bone. 2009;45(3):423-6. doi:10.1016/j.bone.2009.05.012

2. Paranhos-Neto FP, Vieira Neto L, Madeira M, et al. Vitamin D deficiency is associated with cortical bone loss and fractures in the elderly. Eur J Endocrinol.2019;181(5):509-517. doi:10.1530/EJE-19-0197

3. Theodoratou E, Tzoulaki I, Zgaga L, Ioannidis JP. Vitamin D and multiple health outcomes: umbrella review of systematic reviews and meta-analyses of observational studies and randomised trials. BMJ.2014;348:g2035. doi:10.1136/bmj.g2035

4. Oliai Araghi S, van Dijk SC, Ham AC, et al. BMI and Body Fat Mass Is Inversely Associated with Vitamin D Levels in Older Individuals. J Nutr Health Aging.2015;19(10):980-5. doi:10.1007/s12603-015-0657-y

5. Autier P, Mullie P, Macacu A, et al. Effect of vitamin D supplementation on non-skeletal disorders: a systematic review of meta-analyses and randomised trials. Lancet Diabetes Endocrinol.2017;5(12):986-1004. doi:10.1016/S2213-8587(17)30357-1
